# Perceived Benefits and Risks of Social Media: Ethiopian Secondary School Students’ Perspectives

**DOI:** 10.1007/s41347-018-0062-6

**Published:** 2018-06-19

**Authors:** Feyisa Mulisa, Dawit Asrat Getahun

**Affiliations:** Department of Psychology, Bahir Dar University, Bahir Dar, Ethiopia

**Keywords:** Perceived, Social media, Benefits, Risks, Ethiopia

## Abstract

Recent findings show teenagers use social media increasingly in their daily life. There is also a consensus that social media have both pros and perils on students’ academic affairs. The objective of this study is to assess how Ethiopian secondary school students perceive the benefits and risks associated with social media use. Data were collected from 353 secondary schools students through self-reported questionnaire. The findings reveal that the leading perceived benefits of social media are recreational and relational purposes. The role social media play in students’ academic activities is likely less considered. As a pilot study in a developing country, this study may raise awareness of schools, teachers, and parents about the benefits and risks of using social media for teenager students. Thereby, there will be future researches that enable them to effectively monitor their students to use social media primarily for academic purposes.

## Introduction

As findings from various studies show, the use of social media is increasingly becoming popular among the youths worldwide due to their interactive features (Ahn 2011; Carter 2013; Lev-on 2017; Reid and Weigle 2014; Tartari 2015; Williams and Ricciardelli 2014). Social media constitute websites that are designed to enable users to share information, exchange ideas, and participate in content modification online. These involve the platforms Facebook, YouTube, Twitter, Instagram, LinkedIn, Google^+^, and other similar interactive websites. They are widely in use among public services and students in different levels of education (Dhir and Tsai 2017). Reports indicate that the number of users of social media is increasing from time to time and exceeding billions. In 2015, Facebook alone reported that there were 1.49 billon customers that had an account on the platform (Błachnio et al. 2016; Kaya and Bicen 2016). Teenagers are among the prolific users of these social media around the world (Ahn 2011; Mazman and Usluel 2010; Williams and Ricciardelli 2014). The fact that social media platforms are cheaper, user-friendly and more interactive than other modes of communication channels, and openness to everyone (Khan et al. 2014; Kokkinos and Saripanidis 2017) has contributed to the rapid expansion of users. In Ethiopia, although no study has been conducted on the trends of social media use, it is believed that a large number of teenager students are active users. Given that large numbers of social media user, teenager students are coming on board; it seems worthwhile to be concerned about benefits and risks of the users. Hence, the primary objective of this study is to assess how secondary school students in Ethiopia perceive the *benefits* and *risks* associated with social media use. The study may serve as a pilot study for further extensive studies aimed at devising intervention programs that could maximize the benefits and reduce risks associated with using social media.

Worldwide, scientific evidences show that social media are primarily used for recreational and relational purposes (Barth 2015; Kokkinos and Saripanidis 2017; Manasijevic et al. 2016). The use of these media is believed to have both benefits and risks on teenagers’ well-being, including their safety and academic careers (Barth 2015; Jung et al. 2017; Lev-on 2017; Tartari 2015; Tsitsika et al. 2014). To start with its positive aspects, there are studies that highlight the roles of social media in building positive personality traits and increasing the academic opportunities for teenagers (Akcaoglu and David 2016; Kaya and Bicen 2016; Lee and Horsley 2017; Marino et al. 2016). A study by Lee and Horsley (2017), for example, reported that using Facebook among teenagers facilitates the development of six important personality traits: competence, confidence, connection, character, compassion, and contribution, which are bases for students’ better academic performance. Likewise, there are findings that show social media can contribute to the social and emotional maturity of teenagers (Behler 2017; Błachnio et al. 2016; Lee and Horsley 2017). In addition to their contribution to the positive teenagers personality development, through closer monitoring by parents and teachers, social media can certainly contribute to students’ academic progress (Ahn 2011; Akcaoglu and David 2016; Lambic 2016; Manasijevic et al. 2016). Students can share academic information, give and receive academic scaffoldings, and get connected to each other through social media platforms. A study by Akcaoglu and David (2016) further shows that social media can play considerable role to engage students in learning processes, feeling closer to the given course contents, and perceiving their instructors as more involved. Last, social media also have a potential to facilitate collaborative learning among the students and with faculty (Sharma et al. 2015) and motivate students to learn at their own pace and place (Carter 2013; Lambic 2016).

Nonetheless, social media become increasingly being used for educational purposes (Delcore and Neufeld 2017); some authors firmly emphasize the risks related to their use. For example, there are various undesired effects and uncertainties associated with excessive use of social media such as peer humiliation, cyberbullying, depression, isolation, and academic fluctuations (Kokkinos and Saripanidis 2017; Patton et al. 2016; Tsitsika et al. 2014; Woods and Scott 2016). There are also studies that claim social media have significant negative impacts on students’ educational, emotional, and psychological well-being particularly for teenagers (Ahn 2011; Jung et al. 2017; Lev-on 2017; Tartari 2015; Tsitsika et al. 2014). Comparing the benefits of social media with their risks, some authors argue that their disadvantages are far greater than their advantages (Ho et al. 2017; Lev-on 2017; McCrae et al. 2017; Sarabia and Estevez 2016; Young et al. 2017). These risk behaviors can be viewed from three points of view: personal, academic, and emotional (Ahn 2011; Lev-on 2017; Smith et al. 2017). The personal risks that frequently happen to teenager students on social media involve uploading unintended information in certain contexts; uploading negative information about oneself and the others; hacking others’ accounts and being hacked; and using information for retaliation purposes, privacy problem, safety, and psychological well-being of teenagers (Ahn 2011; Błachnio et al. 2016; Lev-on 2017; Smith et al. 2017; Woods and Scott 2016).

Academically, the excessive use of social media has counter effect on the students’ academic pursuits by diverting their attention from education-oriented activities to recreational-oriented activities (Cassidy 2006; Junco 2012b; Tsitsika et al. 2014). Emotionally, the extensive use of social media can reduce peer interactions and may lead users to feel loneliness overtime and lead teenagers develop distorted image of self (Błachnio et al. 2016). Instigated by appearances of other users in the platforms, teenagers may enter into comparing their own physical appearance with those of other peers, which may inevitably cause dissatisfaction with one’s own standard of beauty (Błachnio et al. 2016; Ferguson et al. 2014; Reid and Weigle 2014). This may eventually lead teenagers to emotional depression and social withdrawal (McCrae et al. 2017; Tartari 2015; Woods and Scott 2016). Furthermore, if teenagers are obsessed with their own physical appearance, they may develop narcissistic personality disorder (Kaya and Bicen 2016). Research findings further illustrate that due to addictive social media use, there are major mental health problem, sexting, and suicide among adolescents (Luxton et al. 2012; Reid and Weigle 2014).

Although there are several problems associated with social media use, the benefits of social media have well-considered for educational purposes. For example, it was reported that social media is one of the most effective ways to teach students and influence their achievement (Akcaoglu and David 2016; Kaya and Bicen 2016; Lee and Horsley 2017). In particular, Kaya and Bicen (2016) reported that, if properly managed, social media can help students to achieve better grades. In addition, in the twenty-first century, online professionalism is also a priority to teachers who use Facebook (Prescott 2014). With mindfulness of its side effects, if properly managed, social media may boost students’ academic success and teachers’ professionalism. Accordingly, students and other stakeholders may be benefited from social media for better results. Having considered that social media have both benefits and risks on teenager students in practical terms, we were concerned with how teenager students perceive the benefits and risks of social media use. Therefore, a pilot study of how teenagers perceive the benefits and risks of using social media in the context of developing countries would be worth doing. Hence, the primary objective of this study is to assess how teenager students in secondary school of Ethiopia perceive benefits and risks associated with using social media with intention to offer empirical evidences. As a guide to the study, the researchers formulated the following research questions: What are the perceived benefits associated with the use of social media for teenager students? What are the perceived risks associated with the use of social media for teenager students?

## Methods

### Participants

The participants of the study were selected from five secondary schools located in central Ethiopia which were assumed to have considerable number of social media users. In the five schools, there were 5879 (2848 males and 3031 females) students. A quota of 100 students was allocated to each of the schools constituting a total of 500 students and systematic sampling method was used to select the participants from each school. This number constituted the preliminary sample taken for the study. As the focus of the study was on the social media users, a preliminary inquiring question was included in the data collecting tool to identify users and non-users. Out of the 500 participants included in this study, 353 participants were found to be active users of social media (have a personal account on Facebook, Twitter, and/or other popular social media sites and have a login frequently). The final analysis is done based on responses from these participants. The participants ranged in age from 14 to 19 with a mean age of 17.97. In terms of sex, 164 of them were males and the rest 189 of them were females.

### Data Collecting Tools

**Fig. 1:**
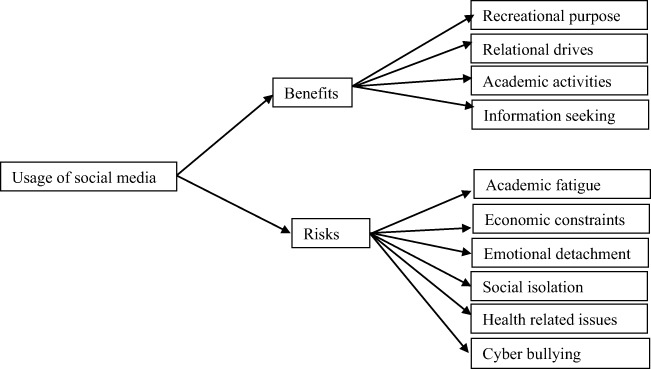
Proposed research patterns

Data were collected using a questionnaire developed by the authors. Since a ready-made measure of social media benefits and risks developed in the context of the study area was not available, the researchers framed eight open-ended questions and administered to 30 students in a surrounding school. The questions required the pilot participants to check the social media sites they use among a list presented, to state their reasons for using social media sites, the activities they engage in when using social media sites, and the benefits they think they get and the risks they think the use of social media sites have brought to them. This enabled the authors to get a range of ideas that can be structured in a closed ended format for examining benefits and risks of using social media. From closer examination of responses to open-ended questions, the authors observed the perceived benefits of social media that can be classified into four categories (recreational, relational, academic, and information seeking) as illustrated in Fig. 1.

There were also six categories of risks associated with using social media sites (academic-related, economic-related, emotion-related, sociological, health-related, and cyberbullying). The final questionnaire was structured to have 11 major sections which focus on users’ behavior (sites they usually use, frequency of use, duration of use per day, the types of activities they perform in using the sites), purpose/reasons for using the sites, the number of connections they have, the whereabouts of the connections (local or abroad), level of personal acquaintance to their connections, the time they spend for academic tasks, the perceived benefits, and perceived risks of using social media sites. For the purpose of the present research report, we focused on data on time spent for academic works and social media sites, frequency of use of social media sites, and perceived benefits and risks of social media use.

The section used for measuring perceived benefits of using social media was composed of 20 items categorized into four subscales: Perceived recreational benefits was measured using five items with Cronbach alpha (0.790); perceived relational benefits was measured using five items with alpha (0.901); perceived information seeking benefits was measured using four items with alpha (0.870); and perceived benefits for academic tasks was measured using six items with alpha (0.923). A five-point rating scale ranging from 1 (representing no benefit of using social media for the mentioned item) to 5 (representing a great deal of benefit of using social media for the mentioned item) was used.

The section meant to measure perceived risks is composed of 23 items measuring six dimensions: Economic constraint was measured using four items with alpha (0.810), academic fatigue was measured using five items with alpha (0.791), emotional detachment was measured using four items with alpha (0.920), social isolation was measured using four items with alpha (0.899), health-related issues with five items alpha (0.796), and cyberbullying was measured using three items with alpha (0.833). Similar to the section meant to measure perceived benefits, five point rating scale ranging from 1 (representing low level of risk) to 5 (high level of risk) was used. In addition to the structured ratings, open-ended questions were added following each of the subscales measuring perceived benefits and perceived risks. The participants were asked to point out further benefits they have received and if they encountered risks from using social media platforms. The data collected through open-ended questionnaire were integrated into analysis using concurrent triangulation design approach (Creswell 2009).

## Results

As the main objective of this study was to assess the teenager students’ perceptions of benefits and risk factors associated with social media use, descriptive statistics such as means, ranking, and percentages were used to make meaning out of the collected data. Results revealed that students likely spend more time on social media per day than on academic activities (Table 1). In particular, the duration of time students spend on Facebook alone seems to be equal with the time spent on academic activities. We also tried to find out if students have to primarily use social media for educational purposes; however, the mean rating of the benefits of social media for academic purposes was ranked the last. Considerable number of students seems to use more than one social media platforms at a time (Table 2). This likely increases the frequency of login into social media accounts and decreases the time of academic engagement.

**Table 1:** The time that students spend on social media and academic activities per day

	Facebook	Twitter	Google+	YouTube	Myspace	Academic works
> 3 h	8.5%	3.7%	0.6%	6.1%	0.00%	17.7%
2–3 h	8.8%	4.9%	0.6%	3.1%	7.1%	16.2%
1:30–2 h	5.5%	0.00%	3.10%	6.1%	14.3	5.9%
1–1:30 h	21.2%	2.4%	16.7%	19.0%	7.1%	24.8%
30–60 min	27.3%	31.7%	29.6%	24.5%	24.3%	10.9%
< 30 min	28.8%	57.3%	49.4%	41.1%	75.1%	14.5%
Mean duration/day	2.62 h	1.74 h	1.78 h	2.24 h	2.07 h	2.64 h

**Table 2:** The nature, frequency, and academic activities students engaged per day

	Active users	Once (%)	Twice (%)	Three times (%)	Four times (%)	Five and more times (%)	Frequency of use/day
Facebook	327	42.2	22.9	16.8	2.8	15.3	2.26 times
YouTube	161	67.3	19.2	5.8	1.9	5.8	1.66 times
Google+	156	64.6	18.0	12.4	2.5	2.5	1.60 times
Twitter	83	78.3	9.6	6.0	4.8	1.2	1.41 times
Myspace	18	88.9	11.1	0.00	0.00	0.00	1.31 times
Academic chores	352	46.02	7.39	18.8	13.6	14.2	2.16 times

### Perceived Benefits of Using Social Media Sites

The mean values of perceived benefits of social media for secondary school students have been computed and presented in ranking (Table 3). The result revealed that students more likely to perceive social media as tools for recreational and relational purposes. For students’ academic activities, the benefit of social media was ranked last and given a mean value below average. Perhaps these results may be a sign that students largely use social media for recreational and relational purposes but not for the academic purposes. Indeed, among the four subscales delineated to measure the benefits of social media, only the academic benefits of social media section was observed below the mean value.

**Table 3:** Descriptive values of students’ perceived benefits and risks of social media usage on five scales measurements

Perceived	Scales	*N*	Mean	SD	Max	Min	Rank
Benefits							
	Recreational purpose	346	19.65	1.29	25	5	1st
	Relational drives	348	17.43	2.43	25	5	2nd
	Information seeking	348	13.78	2.65	20	4	3rd
	Academic activities	347	14.97	2.98	30	6	4th
Risks behaviors							
	Economic constraints	349	18.02	0.69	20	4	1st
	Academic fatigue	345	19.81	1.97	25	5	2nd
	Emotional detachment	347	14.13	1.35	20	4	3rd
	Social isolation	348	13.32	2.12	20	4	4th
	Health-related issues	346	10.56	1.03	25	5	5th
	Cyberbullying	349	4.68	0.65	15	3	6th

### Perceived Risks of Using Social Media Sites

To measure the perceived risks of using social media, the mean values of six dimensions in the subscales were computed and ranked. The mean ranking revealed economic constraint as the primary risk related to using social media, which was raked the first. As the participants are teenager students who are economically dependent on parents, there may be recurring conflicts on the costs incurred in social media, which could gradually trigger teenagers’ emotional detachment from parents. On the other hand, cyberbullying was the last ranked among risks associated with using social media. From the responses obtained through an open-ended question, the participants also pointed out risks associated with using social media such as failure to do homework, wasting of time and poor preparation for exams, poor concentration on academic issues, absenteeism from school, developing many culturally inappropriate behaviors, addiction to social media usage, and health-related problems (such as sleep disturbance and eye burning).

## Discussion

This study investigated the perceived benefits and risks associated with using social media among teenager students in Ethiopia. Based on the findings, teenager students used social media primarily for recreational and social networking purposes. The academic value of social media, which is basically vital for students, was likely less considered. There were many considerable risks associated with social media use among teenager students. Mainly, risks such as financial constraints and academic fatigue were among the top-ranked threatening risks to the students. This finding is virtually consistent with majority of previous studies that highlighted social media are widely used in the areas of recreational and relational purposes than in the academic settings (Akcaoglu and David 2016; Balakrishnan and Lay 2016; Cheung et al. 2011; Dondlinger et al. 2016; Kaya and Bicen 2016; Kümpel et al. 2015; Manasijevic et al. 2016). Notwithstanding that it requires further extensive investigations, in reference to literatures in the western world such as (Carter 2013; Ho et al. 2017; McCrae et al. 2017) in Ethiopia, the rate of cyberbullying among secondary school was relatively at a premature stage. But this does not mean that there is no cyberbullying and may not be increased in the near future. Therefore, it would require continuous follow-up and ongoing research to determine the magnitude of cyberbullying.

Despite it was less considered, there were also evidences that indicate social media was jeopardizing students’ academic achievement through disturbing sleeping programs, disrupting study times, distracting concentration on academic issue, reducing preparation for exam, failing to perform homework on time, and causing absenteeism from school, which reinforces previous findings (Balakrishnan and Lay 2016; Cassidy 2006; Junco 2012a; Tsitsika et al. 2014). Given that students have received limited academic benefits from using social media, there may be profuse potential risks associated with intensive using of these platforms. These risks may range from developing many culturally inappropriate behaviors to health problems and from emotional detachment from family to assigning less time for academic activities. For example, if a mere emotional detachment between teenagers and their families occurred, the chance of the family to involve in their children’s academic pursuits, identity formations, and nurturing socially acceptable behaviors would be reduced. This, in turn, might unenthusiastically affect both the academic and psychosocial development of the teenagers. Furthermore, recurring economic constraints, social isolations, emotional detachment from family members, and occasional cyberbullying can gradually nurture mental health problems. This finding is analogous with previous findings that reported intensive use of social media often associates with mood disturbances, depression, suicidal, and sleep disturbance (Seo et al. 2017; Smith et al. 2017; Woods and Scott 2016). Beside the health problems and academic challenges, in the contexts where Wi-Fi networks rarely available, using these media sites could be associated with high cost of air expense that would need further intervention. This might eventually trigger conflict between the parents and teenager students and reduce the emotional bonds between the teenagers and their parents, which was previously stipulated by (Beyens et al. 2016; Błachnio et al. 2016; Reid and Weigle 2014).

School psychologists can, therefore, help teenager students through launching guidance sessions that enable teenager students from posting, sharing, and exchanging private and confidential contents on social media. Vulnerability to such behaviors may one day cause online harassment and even lead to depression and suicide (Ho et al. 2017). School, as a system, would plan ahead to protect children from inappropriate online interaction and cyberbullying. This might play fundamental role in channeling the uses of social media from recreational purposes to academic-oriented activities. It is possible to change the use of social media from recreation purposes into educational activities through setting up course-specific social media pages and linked blog or multi-author blogs that could enrich the students’ learning. These academic-led social media pages should orient at providing students with contents of subject matters, learning resources, and current commentaries on specific subject matters. Further, academic discourses and participatory instructional designs can be achieved covering a simple communication of topical themes to the revision of curriculums. The parents can play key roles by establishing systems of monitoring and discussing the right ways to use social media honestly. Working in collaboration, the risks associated with using social media might be reduced and the benefits of social media for academic purposes might be increased for secondary school students.

In educational settings, the benefits of social media would likely become greater than associated risk factors (Akcaoglu and David 2016; Lambic 2016; Manasijevic et al. 2016); however, in the current situation in Ethiopia, high school students have placed the importance of social media for academic purpose in the last rank. An explanation for these features may be the initial adoption of social media use for recreational purposes that may matter their current roles for academic activities. Finally, we would like to declare that there are several limitations to this study. The study recruited small sample size, so that generalization of the result may not be possible. There is also a failure to consider whether intensive use of social media has strong relationship with students’ actual academic performance. In addition, we were not able to determine the amount of time teenager students had been spending on other leisure activities before the opening of social media to be sure about the magnitude of social media use today.

## Conclusion

This study was about the perceived benefits and risks of social media use among teenager students in Ethiopia. As seen in the results, social media were primarily used for entertainment and social networking purposes, rather than academic values. In educational settings, they may have no more values than diverting students’ attention from mainstream academic activities. This is due to the fact that social media were extensively used for the recreational purposes, giving limited academic benefits for students. Therefore, acknowledging the recommendations of Seo et al. (2017) who argued for restriction of using electronic media after bedtime and Len-ríos et al. (2016) that stressed the roles of parents in guiding the use of social media, we have no firm stand against the use of social media for academic purposes. Instead, looking for how social media could become rewarding tools for the academic purposes will be prioritized. But strong watchfulness would be taken by the teachers, parents, and school psychologists for the well-being of the learners. With vigilant control, monitoring, and guidance on what to use and how to use, social media may contribute something positive to students’ learning. Additionally, risks associated with using social media can be reduced by training students how to use their time effectively. The future research directions will be on how social media could be exhaustively utilized for educational purpose and enhance students’ learning.
